# Collapse of a Marine Mammal Species Driven by Human Impacts

**DOI:** 10.1371/journal.pone.0043130

**Published:** 2012-09-19

**Authors:** Tero Harkonen, Karin C. Harding, Susan Wilson, Mirgaliy Baimukanov, Lilia Dmitrieva, Carl Johan Svensson, Simon J. Goodman

**Affiliations:** 1 Swedish Museum of Natural History, Stockholm, Sweden; 2 Department of Marine Ecology, Gothenburg University, Göteborg, Sweden; 3 Tara Seal Research Centre, Killyleagh, County Down, United Kingdom; 4 Institute of Hydrobiology and Ecology, Almaty, Kazakhstan; 5 Institute of Integrative and Comparative Biology, University of Leeds, Leeds, United Kingdom; Texas A&M University-Corpus Christi, United States of America

## Abstract

Understanding historical roles of species in ecosystems can be crucial for assessing long term human impacts on environments, providing context for management or restoration objectives, and making conservation evaluations of species status. In most cases limited historical abundance data impedes quantitative investigations, but harvested species may have long-term data accessible from hunting records. Here we make use of annual hunting records for Caspian seals (*Pusa caspica*) dating back to the mid-19^th^ century, and current census data from aerial surveys, to reconstruct historical abundance using a hind-casting model. We estimate the minimum numbers of seals in 1867 to have been 1–1.6 million, but the population declined by at least 90% to around 100,000 individuals by 2005, primarily due to unsustainable hunting throughout the 20^th^ century. This collapse is part of a broader picture of catastrophic ecological change in the Caspian over the 20^th^ Century. Our results combined with fisheries data show that the current biomass of top predators in the Caspian is much reduced compared to historical conditions. The potential for the Caspian and other similar perturbed ecosystems to sustain natural resources of much greater biological and economic value than at present depends on the extent to which a number of anthropogenic impacts can be harnessed.

## Introduction

High removal levels of keystone species may push ecosystems into new equilibria from which they are unlikely to return to historical states [1]. In marine ecosystems such regime shifts often result from unsustainable harvesting of commercially important fish [1], [2], [3] or marine mammal species [4]. Determining the past role of such populations can have important implications for reconstructing the historical state of ecosystems in terms of the biomass concentrated at different trophic levels, help with understanding long term human impacts, and provide goals for restoration and management [1], [5]. Demographic history is also vital for conservation evaluations since the rate of decline is one of the main criteria used in placing taxa in International Union for the Conservation of Nature (IUCN) threat categories [6]. In contrast to most species, harvested species and populations may have time series of hunting or catch data. In this paper we reconstruct the historical abundance and demography of Caspian seals (*Pusa caspica*) based on exceptionally complete hunting records spanning 140 years from the 1860s to the late 20^th^ century. We chart a catastrophic decline in Caspian seals, primarily driven by over-harvesting, and discuss the implications for the Caspian ecosystem and the current conservation status of the species. Our approach should be applicable for analyses of histories for other key species where some current census, harvesting and life history data are available, and therefore a tool for assessments of species against IUCN threat criteria and examining historical changes in ecosystem structures.

Caspian seals are endemic to the Caspian Sea, and have been isolated since diverging from the ancestral *Pusa* genus around 1.3 million years ago [7]. They are one of the main large piscivores in the Caspian and large-scale changes in their abundance may therefore impact the structure of the whole ecosystem. The seals range throughout the entire Caspian Sea, which covers an area of 393,000 km^2^ [8]. The northern ice fields constitute the critical breeding habitat, where pups are born at the end of January to the beginning of February, and weaning after 4–5 weeks [9]. Ice coverage has gradually diminished over the past three decades [10], [11] due to climate warming, and the north-eastern part of the ice-field also overlies one of the world's largest oil fields, which is currently being developed for exploitation. Other issues currently considered as threats to the population include unsustainable levels of hunting and mortality from fisheries by-catch, mass mortalities due to canine distemper virus (CDV), habitat loss and disturbance from industrial development, and possible low prey abundance owing to over-fishing and recent invasion of the Caspian by the comb jellyfish *Mnemiopsis leidyi* [12].

The earliest known evidence for utilisation of Caspian seals by humans dates to around 20,000 years BP in northern Iran [13]. Significant commercial hunting started as early as 1740, and average annual harvests exceeding 100,000 were reported from at least since 1800 [14], [15], [16]. These numbers indicate that Caspian seals were once abundant, but aerial surveys during the 2005 and 2006 pupping seasons showed a decline to around 21,000 breeding females [17].

In this paper we use the unique and extensive hunting data for Caspian seals to reconstruct the minimum population sizes that could have sustained the recorded hunting pressure over the past 140 years up to the year 2005, when an estimate of pup production was determined from an aerial survey of the breeding population on the ice [17]. We then discuss how changes in abundance of seals may have affected the Caspian ecosystem and vice versa. We consider how the relationship between the seal population and the overall Caspian ecosystem may have altered over this period and consider prospects for recovery of this and other depleted seal populations.

## Results

### Changes in population size

**Figure 1:**
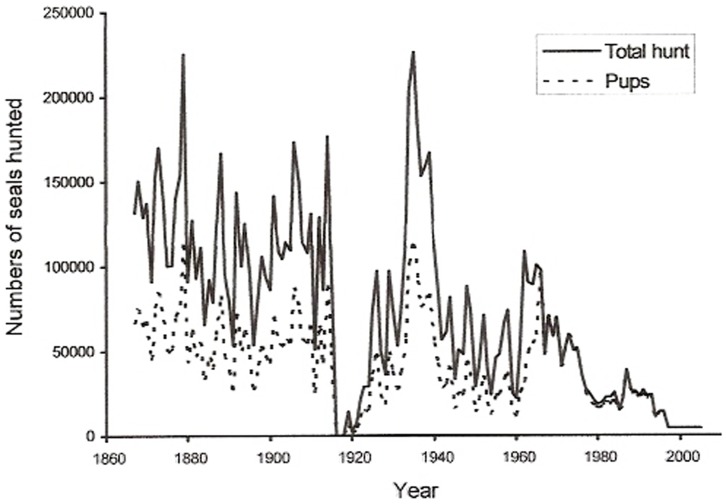
Total registered harvest of Caspian seals (solid line) and the number of pups (dashed line) for the period 1867–2005. Based on published hunting records [14], [15], [16]. Data for recent years are derived from Russian Federal Fisheries Agency reports [29], [30].

**Figure 2:**
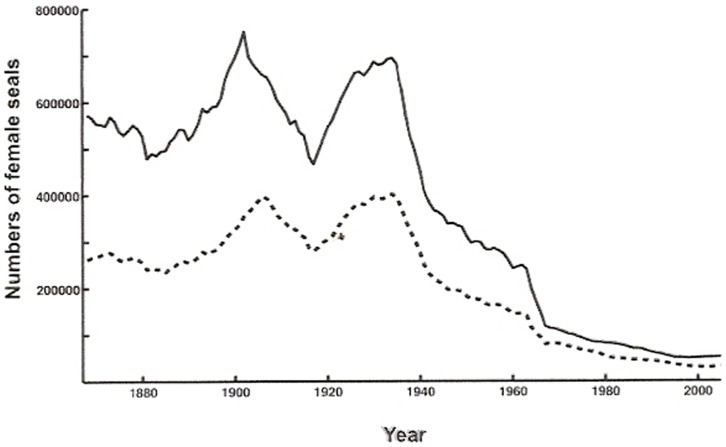
Estimated minimum total female population size (solid line) and the number adult females (dashed line) in the Caspian for the period 1867–2005 as based on historical hunting records (**Fig. 1**). The hunt during the 1960s led to a rapid decline in population size.

**Figure 3:**
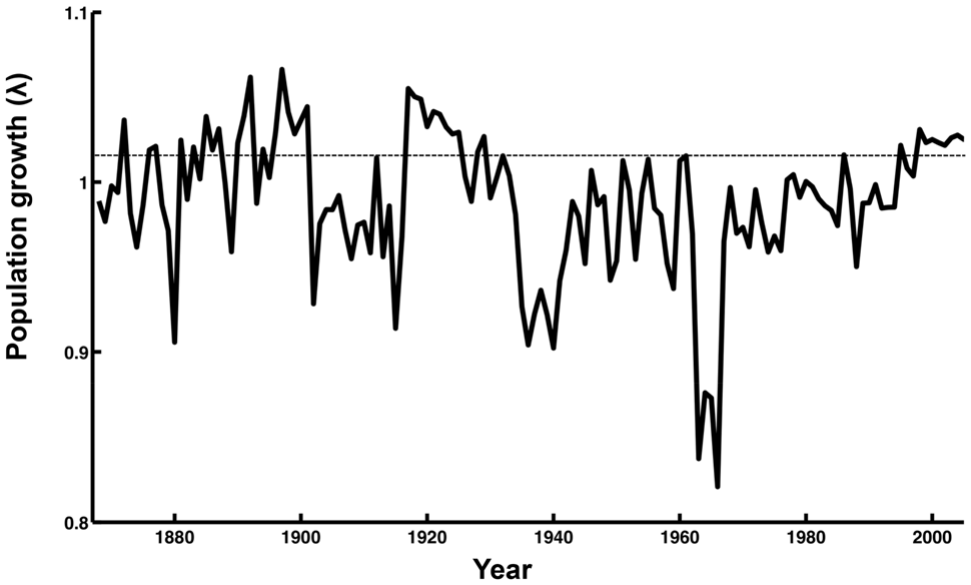
The population growth rate of the Caspian seal population from 1867 to 2006 has fluctuated significantly because of the variable hunting pressure.

Using an age-structured projection model (eqns 1–2 3) and the annually recorded harvest (Fig. 1) of Caspian seals over the period 1867–2005, we estimate the minimum initial female population size in 1867 at 572,800 females, of which 245,830 were breeding (Fig. 2). Given this starting point we estimate about 354,210 females in 1945 of which 193,140 were breeding, and 30,200 in 2005, where the 21,000 breeding females produced the same number of pups, which was approximately the number estimated from the survey in 2005. In simulations employing 20% lower and higher pup survival rates, as a test of the sensitivity to estimates of pup mortality, the estimated population sizes in 1867 were 510,400 and 676,700 females, respectively. Since the sex ratio in Caspian seals is close to parity [9], total initial population size was in the range from 1.0 to 1.6 million seals. Mean population growth was 0.983 for the entire period, using the average juvenile survival rate (Fig. 3), and between 0.982 and 0.986 for low and high pup survival rates, respectively. The population was reduced by about 66% between 1867 and 1964, and by a further 73% between 1965 and 2005 (Fig. 3).

### Changes in population structure

**Figure 4:**
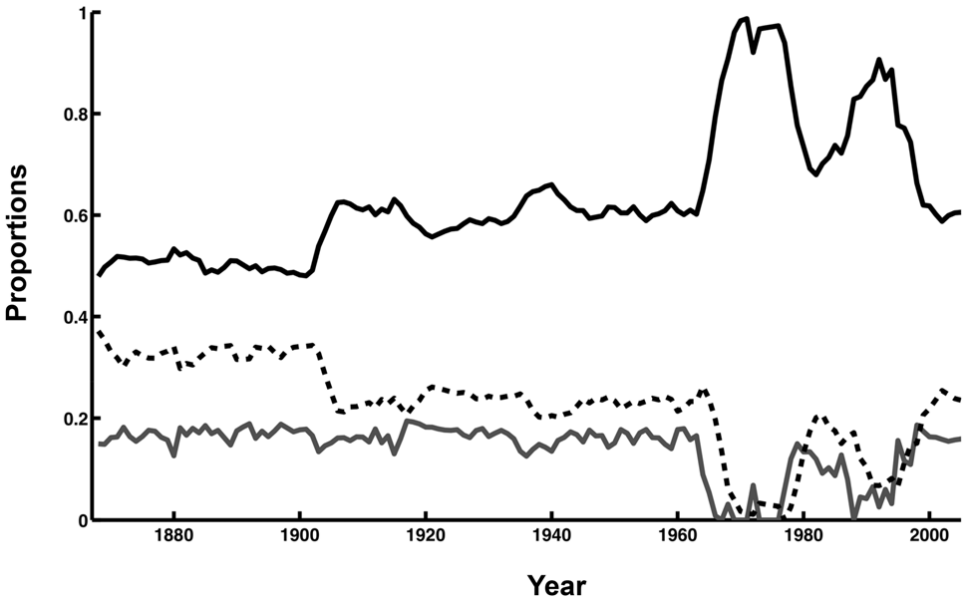
Temporal changes in age structure before pupping of the Caspian seal population. The skewed age structure is mainly due to hunting mortality and, sterility in the 1960s and 1970s. Adults = solid line, sub-adults = dashed line, yearlings = solid grey line.

The intrinsic population growth for the period 1867–1964 was 1.10. However, due to intense hunting there were great fluctuations in the population structure and the realised population growth rate during that period (Fig. 4). A lower figure for the instrinsic population growth for the period 1965 to 2005 was assumed to allow for reported lower fertility due to OC contamination (Table 1). At the estimated population structure in 2005 the 21,000 pupping females in 2005 would represent 20% of the total population size, which would therefore be about 104 thousand seals. Hunting reduced the simulated mean population growth to 0.971 (Fig. 3). Since hunting after 1965 up until the early 1990s was consistently high and focussed on pups, the age structure in the population during this period was strongly skewed towards adults (Fig. 4). Hunting was reduced in the mid 1990s, resulting in an age structure by 2005 which is close to initial conditions in the 19^th^ century (Fig. 4.).

**Table 1:** Vital rates for Caspian seals during the periods 1930–1964 and 1965–2005.

Variable	Description	1867–1964	1965–2006
*F*	Adult fertility	0.94/2	0.7/2
*p*_p_	Pup survival	0.54–0.67–0.80	0.36–0.45–0-54
*p*_s_	Sub-adult survival	0.90	0.90
*p*_a_	Adult survival	0.97	0.97
*m*	Age at sexual maturity	5	5

Three pup survival rates, i.e. 0.8*p*_p_, *p*_p_, or 1.2*p*_p_, are used to attain a realistic span for population size in 1867.

### Generation time

Using the equation 4, the generation time for Caspian seals, measured as the mean age of females giving birth to a cohort, is 18–22 years for adult survival rates ranging between 0.95 and 0.97, respectively.

## Discussion

### Human impacts on seal populations

Human exploitation of pinnipeds has resulted in the extinction of the Caribbean monk seal (*Monachus tropicalis*), the Japanese sea lion (*Zalophus californianus japonicus*) and the extirpation of the Faroese harbour seal (*Phoca vitulina*) [12].

Grey seals (*Halichoerus grypus*) were extirpated from the European mainland North Sea coast in the Middle Ages, from the Skagerrak in the 1750s and the Kattegat in the 1930s [18]. A combination of hunting and other human impacts brought northern elephant seals (*Mirounga angustirostris*) to the brink of extinction [19], and have severely depleted populations of Mediterranean monk seals (*Monachus monachus*) [20], and Hawaiian monk seals (*M. schauinslandi*) [21]. Detailed historical hunting records are lacking for many formerly depleated pinnipeds e.g. Antarctic fur seals (*Arctocephalus gazella*), but such data are available for the Northern fur seal (*Callorhinus ursinus*), Saimaa ringed seal (*Pusa hispida saimensis*), Baltic ringed seal (*Pusa hispida botnica*), Baltic grey seal and the harbour seal (*Phoca vitulina vitulina*) in the Wadden Sea, Kattegat and Skagerrak [22], [23], [24], [25], [26]. Analyses of these hunting records documented collapses in all populations, which were depleted to about 5–10% of pristine abundances before protective measures were taken. The very detailed hunting records for Caspian seals enables a more thorough analysis where we find that numbers of breeding females have decreased from a minimum of 245,800 in 1867 to around 21,000 in 2005, which is a decrease by at least 90%.

### Population estimates for the Caspian seal

An earlier reported population size estimate of about one million Caspian seals in the beginning of the 20th century [9], are fairly consistent with our results, which suggest a minimum of 1.2 million seals in 1900 (Fig. 2). However, an estimate of about 360–400,000 for total population size and 46,800 for the size of the reproductive female stock in 1989s [9], which is frequently cited in international compilations, deviates substantially from our calculations (Fig. 2). Data from our study suggest a total population size of about 128,000 and 30,000 for the number of reproductive females for 1989. The estimates for 2005 (104,000 in total and 21,000 reproductive females) therefore indicate a 19% and 30% decline in total numbers of seals and reproductive females respectively, from our estimate for 1989.

In the projections of earlier population sizes we systematically used high parameter values that resulted in under-estimations of population sizes in the past – hence we take a conservative approach and estimate minimum population sizes. We also assumed that the 20^th^ century hunt killed equal numbers of males and females, when in reality the hunting on ice was mainly targeted at females and pups, while the hunting in spring and late autumn was focused on adult animals of both sexes [9]. Consequently, the average rate of decline in numbers of breeding females for the period 1965–2005 of 3.0% (λ = 0.97) is probably an under-estimate.

### Biases in hunting statistics

The annual catch in the Caspian sealing industry in the first half of the 20^th^ century was registered at the seal oil processing plant on the NE coast of the Caspian. This registration was probably reasonably accurate, since the annual harvest fluctuated considerably, rarely reaching the set quota [9], suggesting catches were not over-reported in order to meet targets. The early Russian authors suggest that the inter-annual fluctuations in numbers of seals hunted in the 19^th^ and first part of the 20^th^ Century primarily reflect variation in hunting effort and access to seals according to winter conditions [15].

### Factors affecting recovery of depleted pinniped populations

Many depleted pinniped populations have shared common combinations of factors which have driven their decline. Recovery of populations depends on the extent to which threats persist and on ecological changes following declines [27].

Most formerly over-exploited pinniped populations have recovered when hunting ceased. Examples include the northern elephant seal, most species of fur seals as well as populations of harbour seals and grey seals. Consequently, protection from hunting has been the single most important factor allowing recovery of formerly depleted seal populations [22], [23], [24], [25], [26]. However, in some cases (such as the northern fur seal and the Saimaa ringed seal) recovery has been inhibited by a combination of new threats, such as by-catch and food chain alterations, which were probably less important during the hunting era (e.g. [28]).

The collapse of the Caspian seal population was driven by non-sustainable hunting which caused a rapid decline up to the mid 1990s (Fig. 1). The first steps towards species conservation should logically be a moratorium on hunting. However, although the commercial hunt ceased in 1996 as it was considered economically unviable, substantial takes for ‘scientific purposes’ have occurred in the Russian sector in most years since then. Sporadic smaller scale commercial hunting by the Russian Federation resumed in 2003, with takes of around 3–5,000 in some years [29], under annual quotas of 18–20,000 seals (with around 9,000 allocated to Russia) set by The Caspian Bioresources Commission [30].

Static fishing nets, in contrast to active gear [31], pose a serious threat of entanglement to many marine mammals, including juveniles of the critically endangered Saimaa ringed seal and Mediterranean monk seals [20], [28], where it is the single most important factor hampering population recovery. By-catch of Baltic ringed and grey seal pups is also substantial [31]. By-catch of Caspian seals, particularly in illegal sturgeon nets is likely to have been a source of significant mortality for Caspian seals, amounting to several 1,000s of animals per year in recent years [32], [33].

Infectious diseases caused by morbilliviruses have resulted in mass mortalities in seals including Antarctic crabeater seals (*Lobodon carcinophaga*) [34] and European harbour seals [35]. Several thousands of seals washed ashore throughout the Caspian in mass mortalities in 1997, 2000 and 2001. Canine distemper virus (CDV) was identified as the primary cause of the 2000 mortality, and the same virus was characterised in 1997 [36], [37], [38]. CDV is believed to have been recurrent in the seal population during the 1990s [39], although no further mass mortalities have been reported since 2001. Large-scale mortalities of tens of thousands of individuals were also reported from earlier years (1955–56 and 1971 [33], but with no conclusive diagnosis of the cause. The long-term population impacts of such mortalities will depend on the frequency and severity of outbreaks [40], [41]. Such catastrophic events reduce the long-term rate of increase of the population and amplify its variance, both leading to dramatically enhanced risks for extinction [40]. It has been suggested that the Caspian seal CDV outbreaks were facilitated or exacerbated by organochlorine contaminant loads [42] but recent re-analysis of these data found no link between OC levels and CDV mortality [43]. Low fertility in Caspian seals (from the 1960s to 1990s) has also been attributed to organochlorine contaminant loads [42], although the levels in 1997–2001 were mostly lower than those found to cause infertility and chronic disease complexes in Baltic ringed and grey seals [44], [45].

All pinnipeds require land or ice for breeding, moulting and rest. Human disturbance at land sites result in habitat loss for many species of seals, such as Mediterranean and Hawaiian monk seals [20], [11] and protected areas have contributed to the recovery of many populations of seals [25], [46].

For Caspian seals the winter ice-field in the north Caspian is essential breeding habitat for which there is no effective substitute. During the past decade industrial shipping in connection with the oil field exploitation has been transiting areas of ice-breeding habitat, resulting in disruption of breeding colonies and habitat destruction [12]. In addition, many terrestrial haul-out sites occupied historically have been abandoned, a process which is probably due to a combination of human disturbance (particularly from human occupation, fishing, and industrial activities), reduction in total population size, and sea level fluctuations.

If the substantial reduction of the Northern Caspian ice fields over the past decades [10], [11] continues, Caspian seals are likely to become affected by the loss of breeding habitat. Global warming has already resulted in diminishing ice fields and more variable ice covered periods in the breeding habitats of some ice-breeding seal species [27], [47]. Pup production in the southern sub-population of Baltic ringed seals is absent during mild winters and the population growth rate is close to zero [48]. In oceanic habitats, seals may be able to migrate to other ice-covered areas [27], but for land-locked seals migration is not an option.

Unsustainable catches of key species in coastal areas world-wide have led to many marine ecosystem collapses, since depletion of large consumer species can result in cascading effects influencing all trophic levels [1], [2], [3]. In some ecosystems marine mammals have constituted one or several of these key extracted species. In the Caspian two of the key megafauna predator populations, the seal and the beluga sturgeon (*Huso huso*), have been severely depleted, and other ecosystems may point to potential implications. Similar to the Caspian, hunting reduced Baltic seal populations by 90–95% during the 20^th^ century. The reduced seal population initially resulted in a ten-fold increase in populations of cod (*Gadus morhua*), but this was followed by commercial over-fishing of cod, which resulted in increased populations of herring (*Clupea harengus*) and sprat (*Sprattus sprattus*) [4]. Current annual catches of commercial fish species in the Baltic (total about one million tons per year) are close to or beyond the maximum sustainable yields [49]. Baltic ringed seals and grey seals, with current populations sizes of 10 and 30 thousand respectively, are unlikely to attain pre-exploitation conditions of 200,000 and 90,000 individuals [22], since this would require half a million tons of fish per year to sustain populations of these sizes.

Reconstruction of historical population sizes of other pinniped populations point to similar changes in productivity and trophic structure in other ecosystems. McClenachan & Cooper [5] estimated the Caribbean monk seal once numbered approximately 300,000 seals. Such a population would consume substantially more fish and invertebrates than are currently produced in present day Caribbean coral reefs, implying a historical productivity matched only by the most remote & pristine Pacific reef systems today.

The Baltic and Caribbean seal cases may have close parallels in the Caspian. Assuming the mean population size of 1.2 million individuals of average mass 60 kg at the beginning of the 20^th^ century, the total biomass of seals would have fallen from around 72,000 tons, to approximately 6,120 tons by 2005. A seal of the size of the Caspian seal requires a daily intake of 3.7–4.5 kg of fish [50], which would suggest the seal population in 1900 would have required between 1.6 and 2.0 million tons of fish per year to sustain it. Food requirements of the 2005 population of around 100 thousand seals would be only about 150 thousand tons of fish per year. In fact, since Russian literature dating from before our hunting dataset suggests intensive commercial hunting of seals began as early as 1740 [14], it is likely the true pre-exploitation population size would have been even higher than the 1.2 million estimated for 1900, with correspondingly higher food requirements at that time.

The influence of recent changes in ecosystem structure [51], [52] on the potential for recovery of the Caspian seal population must be substantial. All commercially important fish stocks in the Caspian, including Caspian herring (*Alosa caspica*), salmon (*Salmo salar*) and all sturgeon species have collapsed or are overfished [53]. Anchovy kilka (*Clupeonella engrauliformis*) is by far the most abundant and productive fish species in the Caspian Sea, and it has been harvested commercially since 1925 [54]. Catches peaked in the early 1970s at 420,000 tons, followed by a long-term decline due to overfishing and a collapse to about 75,000 tons in 2005 [55]. Although Caspian seals are opportunistic predators, able to take a wide variety of fish species and not just those targeted by commercial fisheries [56], reduction in availability of high-energy prey and an enforced shift to less energy dense prey items could limit the ability of females to breed successfully. The comb jelly fish *Mnemiopsis leidyi*, accidentally introduced in ship's ballast in the late 1990s, competes with many fish species, including kilka, for zooplankton resources and consumes large quantities of fish larvae, including those of kilka [57]. *Mnemiopsis* may therefore be another factor in the decline of kilka. Primary productivity may also have been affected in the mid-late 20^th^ century due to fluctuations arising from the in-flow of nutrients linked to eutrophification and damming of principle rivers entering the Caspian [58].

There is growing evidence from different ecosystems that large scale ecosystem changes may have profound consequences for the possibilities for large marine vertebrates to recover to former population sizes since pristine conditions of the ecosystems cannot be restored [4], [5], and this problem will be especially acute for a species like the Caspian seal which has no possibilities for migration. Reconstruction of potential historical ecosystem states from archive data such as that presented here, could be a valuable tool contributing to a wide variety of impact assessments, setting of management goals, and evaluation of conservation status.

The conservation status of the Caspian seal has been evaluated according to the IUCN criteria A1b: “Population reduction observed, estimated, inferred, or suspected in the past where the causes of the reduction are clearly reversible AND understood AND have ceased, based on … an index of abundance.”, where the rate of decline over the past three generations should exceed 70% to fulfil the status “Endangered”. The generation time in Caspian seals is approximately 20 years, and three generations (i.e. about 60 years) back from 2005 would therefore suggest 1945 as a reference year for evaluations according to IUCN criteria. The projection in Fig. 2 indicates that the number of reproducing females declined by about 90% between 1945 and 2005. Consequently, the species meets the criteria for the endangered category. The species further fulfils criteria A1d (actual or potential levels of exploitation) and A1e (effects of introduced taxa, hybridization, pathogens, pollutants, competitors or parasites) due to threats of catastrophic reduction in stocks of fish prey, disease epizootics, from continued scientific and commercial hunting, and mortality from fishing by-catch. In addition future climate change may reduce or eliminate altogether the seals' ice-field breeding habitat, while oil industry activity is already disrupting breeding colonies. Industrial and urban development and disturbance around the whole Caspian has led to the abandonment of historical haul-out sites.

## Materials and Methods

We estimate the minimum population size of seals that must have been present in the Caspian Sea during the past 140 years to sustain the documented hunt. To perform the modelling we need a) An estimate of current population size, b) Life history data for the Caspian seal, c) Hunting records, and d) A method to project the population in time, requiring e) estimation of the initial population structure in 1867. f) For assesment of the conservation status of Caspian seals according to IUCN criteria we also need to estimate the generation time.

### Population size in 2005

Surveys of the entire pupping ice area in the Northern Caspian Sea were carried out in the last two weeks of February 2005 – 2006 [17]. Pup production estimated from the first survey was around 21,000 pups in 2005 and 17,000 pups the following year. Data from the first survey in 2005 were used as input values for estimates of population size.

### Life history data

Caspian seal life history data share many characteristics with ringed seals (*Pusa hispida*), since the maximum life-span is about 50 years of age, and they mature relatively late [9], [59], with females reported to usually becoming pregnant at 7 years of age, and giving birth at most to a single pup per year [9], [59]. However, ages at first pregnancy as late as 7 have only been reported in seal populations that are food limited [60], [61]. Since age at sexual maturity also changes with the general nutritive condition or density of the population [60], [61], we use an age at first parturition of six years (Smith 1987, Reeves 1998) and therefore of sexual maturity at five years, which will lead to underestimates of the minimum population sizes that could sustain the recorded hunt (we do this deliberately to ascertain minimum estimates). The maximum rate of increase (which also gives us the smallest population size that can sustain a given hunt) in species with life history features similar to the Caspian seal has been shown to be approximately 10% per year (λ = 1.10) [62]. Using this scenario, we estimated and inferred age specific survival and fertility rates (Table 1) for Caspian seals between 1867 and 2005 by adjusting these parameters in the projection matrix to give a long-term intrinsic rate of increase at λ≈1.10. Fertility rates have been reported to be lower since 1964 [32] (Table 1). Using this maximum long-term rate of increase result in estimates of minimum population sizes in the past since lower rates of population increase would require greater population sizes to withstand the recorded hunt,

### Hunting records

A fleet of sealing vessels provided blubber to a processing plant in Fort Shevchenko in the North-eastern Caspian from the mid 19^th^ century until the 1970s. Annual records of the harvest also included data about the composition of the hunt, where females with pups were targeted up to 1965, after which the hunt was focussed on pups (Fig. 1). Annual records of the harvest also included data about the composition of the hunt. These data are likely to represent fairly accurate hunting records, since the pelts were transported to other factories, where records with comparable figures were kept. Sklabinskij [14] reports that large scale commercial hunting began in 1740, and cites average annual harvests of 160,000 seals prior to 1803 without specifying the period. An average annual catch of 104,651 per year is reported from 1824–1867, with 290,000 in 1844 [14], [16]. Until the mid-19^th^ century, seals of all ages and both sexes were taken in summer and autumn on the unpopulated islands of the northern and southern Caspian. This hunt declined by the end of the 19^th^ century, and the hunt turned to targeting females with pups on ice from the last quarter of the 19^th^ century. For 8 years (1933–1940), catches of females and pups were so high (averaging more than 160,000 and increasing to more than 220,000 annually) that this hunting strategy was believed to have been the main cause of the population decline [16]. From 1941 catches were less than 100,000 annually, and after 1965 the hunt was focussed only on pups. For our focal dataset we make use of individual annual statistics available from 1867 onwards (S1) [9], [15], [29], [30].

### A population projection model

Using vital rates (survival and fertility) for Caspian seals given in Table 1 and the recorded hunt (Fig. 1), the initial population size in 1867 can be iterated such that the projection matches observed numbers of pups in 2005, which is equivalent to the number of reproducing females in that year. This allows estimates of changes in the minimum population size to be made for the period 1867–2005.

Based on available data (Table 1, [62], [63], [64]) we assume a stable intrinsic rate of increase at λ≈1.10 at the beginning of the 1860s. We project the Caspian seal population from 1867 onwards to 2005, keeping track of the number of animals in each age class throughout the projection. Since pup survival can vary substantially we investigated effects of low, average, and high levels of pup survival (Table 1) on estimated historical population sizes. However, results in the following refer to the average scenario if not otherwise stated.

In the age-structured population model (Fig. 2), *N*_i_ denotes the number of female seals at the end of the *i*th breeding season, where *i* is age in years, starting at *i* = 0 (new-born female pup) and ranging up to the oldest age class *c*. We assume even sex ratios for all age classes, but only model the female population. We estimate survival and fertility rates for three age groups; pups, sub-adults and adults. The mean age at sexual maturity is denoted *m*. Pups correspond to age-class zero, sub-adults to age-classes one to *m*−1, and adults to age-classes *m* and above.

There are four vital rates in the model: The probability *p*_p_ that a new-born pup survives the first year; the probabilities *p*_s_ and *p*_a_ of sub-adult and adult survival and the probability *f* for a mature female seal to give birth to a female pup.

The number of new-born pups (*N_p_*) is for each year (*t*) estimated by:
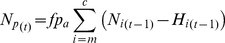
(1)where *f* is the fertility, *p*_a_ adult survival, and the sum gives the number of adult female seals that survived the annual hunt (*N-H*) in the previous year.

The number of sub-adults in each age class was estimated by including both natural and hunting mortality:
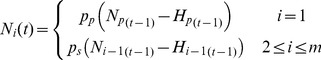
(2)where the number of one-year-olds (i = 1) is given by the pup survival (*p*_p_) multiplied by the number of pups that survived the hunt the year before. *H*_p(*t*−1)_ is the number of pups hunted the year before, and *H*_i−1(t−1)_ the number of sub-adults of age *i*−1 hunted at *t*−1. In an identical manner the number of adults in each age class was estimated by subtracting the seals killed in the hunt and thereafter multiplying the number of survivors with the natural survival probability for adults.
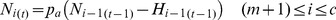
(3)where *p*_a_ is adult survival.

### Initial age structure year 1867

The projection is based on the assumption of a stable age structure, which can be derived from the life history parameters [65]. As the population is projected forward from 1867 to 2005, the annual recorded hunt of adults, sub-adults and pups (Table 1, S1) are removed each year from the population and the resulting age structure is primarily a result of the harvest and the population growth rate. The effect of the assumed initial structure is therefore insignificant for the overall outcome, since the large and biased hunt is the main determinant for the age distribution.

### Generation time

Using the parameter values in Table 1 we create two life-tables, one for each of the periods 1867–1964, and 1964–2005. A life table includes the parameters; *x*, the age, *l*_x_ the proportion of a cohort left in age-class *x*, and *m*_x_ the fecundity of each age class. Using the age structure and fecundity from the life tables of Caspian seals the estimated generation time (*T*), defined as ‘the mean age of females giving birth to a cohort’, is calculated as:
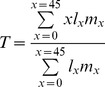
(4)The denominator is equal to the net reproductive rate. In addition, we vary the transition between the adult age-classes (*p*_a_) between 0.95 and 0.97 to obtain a span of probable generation times.

## Conclusions

Our study shows that the collapse in the Caspian seal population was primarily driven by overharvesting. The distribution of the Caspian seal in a completely closed ecosystem, from which individuals cannot disperse to or from adjacent habitats, makes it extremely vulnerable to some or all of the many threats it currently faces, which include mortality caused by hunting and by-catch, reduction in stocks of prey fish and oil industry activities in the ice breeding grounds combined with potential ice-field reductions due to climate change. Until this array of threats can be resolved by the implementation of effective conservation measures, as laid out in the Caspian Seal Conservation Action Plan [66], further rapid declines of this species are likely in the short term.

## Supporting Information

Table S1 **Caspian seal hunting data 1867–2005.**
(DOC)
