# Supplementary material for: Collapse of a Marine Mammal Species Driven by Human Impacts
Source: PLoS One. 2012 Sep 19;7(9):e43130. doi: 10.1371/journal.pone.0043130 (PMC3446954; doi:10.1371/journal.pone.0043130)
Supplement: Table S1 — Caspian seal hunting data 1867–2005. (DOC) [file pone.0043130.s001.doc]

**Supplementary Table S1.**

| **Year** | **Total hunt** | **Pups** | **Reference** |
| --- | --- | --- | --- |
| **1867** | 131700 | 65850 | 15 |
| **1868** | 150900 | 75450 | 15 |
| **1869** | 128700 | 64450 | 15 |
| **1870** | 137000 | 68500 | 15 |
| **1871** | 90500 | 45250 | 15 |
| **1872** | 151800 | 75900 | 15 |
| **1873** | 170500 | 85250 | 15 |
| **1874** | 137900 | 68950 | 15 |
| **1875** | 99700 | 49850 | 15 |
| **1876** | 100900 | 50450 | 15 |
| **1877** | 140300 | 70150 | 15 |
| **1878** | 154400 | 77200 | 15 |
| **1879** | 225100 | 112550 | 15 |
| **1880** | 90700 | 45350 | 15 |
| **1881** | 127400 | 63700 | 15 |
| **1882** | 93200 | 46600 | 15 |
| **1883** | 111200 | 55600 | 15 |
| **1884** | 65700 | 32850 | 15 |
| **1885** | 92400 | 46200 | 15 |
| **1886** | 78900 | 39450 | 15 |
| **1887** | 119600 | 59800 | 15 |
| **1888** | 166800 | 83400 | 15 |
| **1889** | 95700 | 47850 | 15 |
| **1890** | 77900 | 38950 | 15 |
| **1891** | 53500 | 26750 | 15 |
| **1892** | 143600 | 71800 | 15 |
| **1893** | 100200 | 50100 | 15 |
| **1894** | 125200 | 62600 | 15 |
| **1895** | 95600 | 47800 | 15 |
| **1896** | 53200 | 26600 | 15 |
| **1897** | 83800 | 41900 | 15 |
| **1898** | 105700 | 52850 | 15 |
| **1899** | 94500 | 47250 | 15 |
| **1900** | 86100 | 43050 | 15 |
| **1901** | 141400 | 70700 | 15 |
| **1902** | 108600 | 54300 | 15 |
| **1903** | 103900 | 51950 | 15 |
| **1904** | 114300 | 57150 | 15 |
| **1905** | 109400 | 54700 | 15 |
| **1906** | 133300 | 86650 | 15 |
| **1907** | 149800 | 74900 | 15 |
| **1908** | 114100 | 57050 | 15 |
| **1909** | 108000 | 54000 | 15 |
| **1910** | 130700 | 65350 | 15 |
| **1911** | 50800 | 25400 | 15 |
| **1912** | 129000 | 64500 | 15 |
| **1913** | 86100 | 43050 | 15 |
| **1914** | 177000 | 88500 | 15 |
| **1915** | 99700 | 49850 | 15 |
| **1916** |  |  | No data recorded |
| **1917** |  |  | No data recorded |
| **1918** |  |  | No data recorded |
| **1919** | 14000 | 7000 | 9 |
| **1920** | 2000 | 1000 | 9 |
| **1921** | 8000 | 4000 | 9 |
| **1922** | 21000 | 10500 | 9 |
| **1923** | 29000 | 14500 | 9 |
| **1924** | 29000 | 14500 | 9 |
| **1925** | 72000 | 36000 | 9 |
| **1926** | 97000 | 48500 | 9 |
| **1927** | 50000 | 25000 | 9 |
| **1928** | 36000 | 18000 | 9 |
| **1929** | 97000 | 48500 | 9 |
| **1930** | 76000 | 38000 | 9 |
| **1931** | 53000 | 26500 | 9 |
| **1932** | 75000 | 37500 | 9 |
| **1933** | 116000 | 58000 | 9 |
| **1934** | 203000 | 101500 | 9 |
| **1935** | 226000 | 113000 | 9 |
| **1936** | 183000 | 91500 | 9 |
| **1937** | 153000 | 76500 | 9 |
| **1938** | 160000 | 80000 | 9 |
| **1939** | 167000 | 83500 | 9 |
| **1940** | 110000 | 55000 | 9 |
| **1941** | 86000 | 43000 | 9 |
| **1942** | 56000 | 28000 | 9 |
| **1943** | 61000 | 30500 | 9 |
| **1944** | 82000 | 41000 | 9 |
| **1945** | 33000 | 16500 | 9 |
| **1946** | 51000 | 25500 | 9 |
| **1947** | 48000 | 24000 | 9 |
| **1948** | 88000 | 44000 | 9 |
| **1949** | 74000 | 37000 | 9 |
| **1950** | 29000 | 14500 | 9 |
| **1951** | 41000 | 20500 | 9 |
| **1952** | 71000 | 35500 | 9 |
| **1953** | 39000 | 19500 | 9 |
| **1954** | 24000 | 12000 | 9 |
| **1955** | 46000 | 23000 | 9 |
| **1956** | 48000 | 24000 | 9 |
| **1957** | 66000 | 33000 | 9 |
| **1958** | 74000 | 37000 | 9 |
| **1959** | 25000 | 12500 | 9 |
| **1960** | 22000 | 11000 | 9 |
| **1961** | 50000 | 25000 | 9 |
| **1962** | 108300 | 31000 | 9 |
| **1963** | 90400 | 48500 | 9 |
| **1964** | 89000 | 53500.71 | 9 |
| **1965** | 101000 | 59188.17 | 9 |
| **1966** | 97000 | 95870.78 | 9 |
| **1967** | 47600 | 47600 | 9 |
| **1968** | 70200 | 70200 | 9 |
| **1969** | 58600 | 58600 | 9 |
| **1970** | 69800 | 69200 | 9 |
| **1971** | 41400 | 41100 | 9 |
| **1972** | 51200 | 51100 | 9 |
| **1973** | 60000 | 60000 | 9 |
| **1974** | 50100 | 50100 | 9 |
| **1975** | 51400 | 51400 | 9 |
| **1976** | 35200 | 35200 | 9 |
| **1977** | 27400 | 25900 | 9 |
| **1978** | 23600 | 19200 | 9 |
| **1979** | 20500 | 18500 | 9 |
| **1980** | 18300 | 16300 | 9 |
| **1981** | 19800 | 16800 | 9 |
| **1982** | 22700 | 19900 | 9 |
| **1983** | 22800 | 18400 | 9 |
| **1984** | 25500 | 20500 | 9 |
| **1985** | 15000 | 14250 | 9 |
| **1986** | 22000 | 20900 | 9 |
| **1987** | 39000 | 37050 | 9 |
| **1988** | 26000 | 24700 | 9 |
| **1989** | 26000 | 24700 | 9 |
| **1990** | 23000 | 21850 | 29 |
| **1991** | 27000 | 25650 | 29 |
| **1992** | 23000 | 21850 | 29 |
| **1993** | 24000 | 22800 | 29 |
| **1994** | 11000 | 10450 | 29 |
| **1995** | 14000 | 13300 | 29 |
| **1996** | 14000 | 13300 | 29 |
| **1997** | 4500 | 4275 | 29 |
| **1998** | 4500 | 4275 | 29 |
| **1999** | 4500 | 4275 | 29 |
| **2000** | 4500 | 4275 | 29 |
| **2001** | 4500 | 4275 | 29 |
| **2002** | 4500 | 4275 | 29 |
| **2003** | 4500 | 4275 | 29 |
| **2004** | 4500 | 4275 | 29 |
| **2005** | 4500 | 4275 | 29 |
